# Nonelective cesarean section is associated with the prevalence of asthma among Mexican children who attended childcare centers

**DOI:** 10.5415/apallergy.0000000000000144

**Published:** 2024-04-04

**Authors:** Martín Bedolla-Barajas, Jaime Morales-Romero, Ilce Estefanía Contreras-Aceves, Gabriela Gaxiola-de Alba, María del Rocío Estrada-Bedolla, Tonatiuh Ramses Bedolla-Pulido

**Affiliations:** 1 Nuevo Hospital Civil de Guadalajara “Dr. Juan I. Menchaca, Servicio de Alergia e Inmunología Clínica,” Guadalajara, Jalisco, Mexico; 2 Universidad Veracruzana, Instituto de Salud Pública, Xalapa, Veracruz, Mexico; 3 Universidad Michoacana de San Nicolás de Hidalgo, Facultad de Enfermería, Morelia, Michoacán, Mexico

**Keywords:** Allergic diseases, cesarean section, prevalence, transversal study

## Abstract

**Background:**

The cesarean section (CS) mode of delivery can influence the prevalence of bronchial asthma (BA), allergic rhinitis (AR), or atopic dermatitis (AD) by promoting modifications in the infantile microbiome.

**Objective:**

To analyze the prevalence of asthma in children who were born through CS and attended childcare centers.

**Methods:**

The data were obtained through an online survey that was answered anonymously by one of the parents; the survey inquired about the route of delivery of the child and the prevalence of BA, AR, and AD.

**Results:**

A total of 525 children were included. The frequency of births by vaginal, elective CS, or nonelective CS was 34.1%, 37.9%, and 28.0%, respectively, and the prevalence of BA, AR, and AD was 4.8%, 19.8%, and 12.4%, respectively. Multivariate analyses identified nonelective CS as a factor associated with the prevalence of BA (odds ratio: 3.51, *P* = 0.026).

**Conclusion:**

Our study shows that being born through nonelective CS can increase the probability of BA in children who attended daycare centers.

## 1. Introduction

Allergic diseases in children are among the most frequent group of noncommunicable diseases worldwide. Notably, some regions of the world continue to see upward changes in its prevalence. In Mexico, bronchial asthma (BA), allergic rhinitis (AR), and atopic dermatitis (AD) in both school children and adolescents have significantly increased their frequency over a period of approximately 15 years [1-3]. Although the reasons for this trend are still being studied, cesarean section (CS) births may be related. According to the results of the Organization for Economic Cooperation and Development, Mexico ranked among the highest in births through CS (58.6%), followed by Turkey (57.3%) and South Korea (50.6%) in 2019 [4].

Worldwide, when comparing the risk of developing allergic diseases among children born through CS vs those born vaginally, some studies have been able to show an association between the variables [5-8]; but many other conducted studies have not proven any associated factors [9-11]. A relevant point when analyzing the role of birth through CS is considering whether the procedure will be performed electively or not, since the mother’s clinical conditions will be different in each scenario [12].

There are very few studies in Latin American countries and the Caribbean that aimed at evaluating the role played by the route of delivery on allergic diseases despite these regions of the world having the highest numbers of cesarean births (42.8%) [13].

Thus, the objective of our study was to analyze the role played by the CS route of delivery, whether elective or nonelective, on the prevalence of BA in children who attended public daycare centers. A similar target was designed for the AR and AD.

## 2. Methods

### 2.1. Setting and sample size

Through a cross-sectional study, a census was carried out of children who attended 1 of the 12 Child Development Centers of the System for the Family Integral Development (DIF) located in Guadalajara, Mexico. The DIF is a public institution that offers services to children whose parents have to work, which temporarily prevents them from providing care and education. Children aged 6 months to 78 months of age were included. The parents or legal guardians of 650 children were surveyed between May 16 and 30, 2022.

### 2.2. Independent variables

The independent variables were obtained from an anonymous survey that was answered online. This tool took into account the following variables of the child: sex, age in months, weight at birth, duration of breastfeeding, and age at the time of the complementary introduction of food. Parents’ history of smoking and family history of allergic diseases were important factors taken into consideration. The route of delivery was categorized as follows: vaginal route, elective CS, and nonelective CS route (CS during labor, urgent CS, and emergent CS) [12].

### 2.3. Dependent variables

The dependent variables were obtained from the same survey from which the independent variables were obtained. The prevalence of allergic diseases and their symptoms in children were determined from the affirmative answer to any of the following questions:

1. BA: Has your child ever been diagnosed with asthma? (BA diagnosed by a doctor). In the past 12 months, has your child wheezed or whistled in his/her chest?
2. AR: Has your child ever been diagnosed with AR? (AR diagnosed by a doctor). In the last 12 months, has your child sneezed or presented a runny or stuffed nose when he/she did not have the flu or cold symptoms?
3. AD: Has your child ever been diagnosed with AD, neurodermatitis, or eczema? (AD diagnosed by a doctor). In the last 12 months, has your child had an itchy rash?

### 2.4. Analysis

The continuous variables and the free distribution were estimated, with the median and the 25% and 75% percentiles (P_25_-P_75_). Categorical variables were expressed as frequencies and proportions; 95% confidence intervals were estimated for proportions. In the comparison of qualitative variables, the Chi-square test or Fisher exact test was used as necessary. To compare medians, the Mann-Whitney *U* test was used. Multivariate analysis by binary logistic regression was carried out to evaluate the role of the CS route of delivery (independent variable) with the prevalence of allergic diseases (dependent variable). In these regression models, the adjustment variables were sex (male), age ≥60 months, birth weight <2.5 kg, maternal smoking, maternal or paternal atopy, and concomitant allergic disease.

### 2.5. Ethics

This research study was approved by Ethic and Research Committee from Nuevo Hospital Civil de Guadalajara “Dr. Juan I. Menchaca” (no. CEI-00008; Chairperson Aguilar J, MD; July 15, 2021) and by the DIF children’s daycare center director in Guadalajara, Mexico. We obtained the parents’ or legal guardian’s approval of participating in the study by answering the online survey.

## 3. Results

### 3.1. Description of the population

A total of 525 children were included (51.4% female; median age, 50 months), a participation rate of 81%. The children’s median birth weight, duration of breastfeeding, and complementary food introduction were 3.2 kg, 6 months, and 6 months, respectively; 6.1% of the participants had a history of premature birth. About 12.6% of mothers and 22.9% of fathers currently smoke. Also 18.5% of mothers and 10.5% of fathers had a past medical history of atopy.

The frequency of vaginal delivery, elective CS, and nonelective CS was 34.1%, 37.9%, and 28.0%, respectively. On the other hand, the prevalence of allergic diseases and their symptoms were the following: BA and wheezing the previous year, 4.8% and 19.0%; AR and rhinitis the previous year, 19.8% and 57.7%; AD and dermatitis the previous year, 12.4% and 16.6%.

### 3.2. Factors associated with the allergic diseases

Table 1 shows the univariate analyses of the factors associated with the prevalence of allergic diseases. In the case of BA, the associated variables were maternal smoking (*P* = 0.017), maternal history of atopy (*P* = 0.021), and AR (*P* < 0.0001). AR was associated with sex, it was more prevalent in men (*P* = 0.018); this was also shown in maternal (*P* < 0.0001) and paternal (*P* < 0.0001) atopy history, with BA (*P* < 0.0001) and AD (*P* < 0.0001). The associated variables for AD were maternal and paternal history of atopy (*P* = 0.041 and *P* = 0.025, respectively) and AR (*P* < 0.0001). Notably, the route of delivery was not associated with any of the 3 allergic diseases (*P* > 0.05).

**Table 1.** Univariate analysis of factors associated with the allergic diseases

	Bronchial asthma			Allergic rhinitis			Atopic dermatitis		
	No n = 500	Yes n = 25	*P*	No n = 421	Yes n = 104	*P*	No n = 460	Yes n = 65	*P*
Sex, n (%)			0.725			0.038			0.910
Female	258 (51.6)	12 (48.0)		226 (53.7)	44 (42.3)		237 (51.5)	33 (50.8)	
Male	242 (48.2)	13 (52.0)		195 (46.3)	60 (57.7)		223 (48.5)	32 (49.2)	
Age, mo, median (P_25_-P_75_)	50 (36–62)	60 (40-67)	0.077	50 (37–62)	49 (36–65)	0.923	50 (37-62)	51 (37-65)	0.681
Route of delivery, n (%)			0.063			0.926			0.082
Vaginal	174 (34.8)	5 (20.0)		145 (34.4)	34 (32.7)		158 (34.3)	21 (32.3)	
Elective cesarean section	191 (38.2)	8 (32.0)		158 (37.5)	41 (39.4)		167 (36.3)	32 (49.2)	
Nonelective cesarean section	135 (27.0)	12 (48.0)		118 (28.0)	29 (27.9)		135 (29.3)	12 (18.5)	
Prematurity, *n* (%)	30 (6.0)	2 (8.0)	0.659	26 (6.2)	6 (5.8)	0.877	29 (6.3)	3 (4.6)	0.785
Birth weight, kg, median (P_25_-P_75_)	3.2 (2.9–3.5)	3.1 (2.7–3.5)	0.223	3.2 (2.9–3.5)	3.1 (2.8–3.5)	0.315	3.2 (2.9–3.5)	3.3 (2.8–3.6)	0.396
Breastfeeding, mo, median (P_25_-P_75_)	6 (3–12)	6 (3–12)	0.745	6 (3–12)	7 (3–13)	0.467	6 (3–12)	6 (3–18)	0.468
Complementary feeding, mo, median (P_25_-P_75_)	6 (5–6)	6 (5–6)	0.295	6 (5–6)	6 (5–6)	0.754	6 (5–6)	6 (5–6)	0.408
Current smoking, n (%)									
Maternal	59 (11.8)	7 (28.0)	0.017	57 (13.5)	9 (8.7)	0.178	58 (12.6)	8 (12.3)	0.945
Paternal	116 (23.2)	4 (16.0)	0.403	102 (24.2)	18 (17.3)	0.132	105 (22.8)	15 (23.1)	0.964
Familial atopy, n (%)									
Maternal	88 (17.6)	9 (36.0)	0.021	60 (14.3)	37 (35.6)	<0.0001	79 (17.2)	18 (27.7)	0.041
Paternal	52 (10.4)	3 (12.0)	0.738	29 (6.9)	26 (25.0)	<0.0001	43 (9.3)	12 (18.5)	0.025
Comorbidity, n (%)									
Asthma	NA	NA	NA	12 (2.9)	13 (12.5)	<0.0001	21 (4.6)	4 (6.2)	0.535
Allergic rhinitis	91 (18.2)	13 (52.0)	<0.0001	NA	NA	NA	75 (16.3)	29 (44.6)	<0.0001
Atopic dermatitis	61 (12.2)	4 (16.0)	0.535	36 (8.6)	29 (27.9)	<0.0001	NA	NA	NA

NA, not applicable; P_25_-P_75_, 25th percentile–75th percentile.

Table 2 shows that children who presented with wheezing in the previous year were significantly younger (*P* = 0.003), had a higher frequency of maternal smoking (*P* = 0.031), had a greater maternal and paternal history of atopy (*P* < 0.0001 and *P* = 0.018, respectively), and had a higher prevalence of AR (*P* < 0.0001) and AD (*P* = 0.001). Rhinitis symptoms in the previous year were associated with the male sex (*P* = 0.003), younger age (*P* = 0.017), and maternal (*P* < 0.0001) and paternal history of atopy (*P* = 0.017), as well as a higher frequency of BA (*P* = 0.001) and AD (*P* = 0.005). In the case of dermatitis symptoms during the previous year, there was a significant association with age (*P* = 0.037), maternal (*P* = 0.036) and paternal history of atopy (*P* = 0.024), and AR (*P* = 0.002). There was no association between the route of birth and the previous year’s symptoms of allergic diseases (*P* > 0.05).

**Table 2.** Univariate analysis of factors associated with the allergic disease symptoms in the previous year

	Wheezing previous year			Rhinitis previous year			Dermatitis previous year		
	No n = 425	Yes n = 100	*P*	No n = 222	Yes n = 303	*P*	No n = 438	Yes n = 87	*P*
Sex, n (%)			0.227			0.003			0.768
Female	224 (52.7)	46 (46.0)		131 (59.0)	139 (45.9)		224 (51.1)	46 (52.9)	
Male	201 (47.3)	54 (54.0)		91 (41.0)	164 (54.1)		214 (48.9)	41 (47.1)	
Age, mo, median (P_25_-P_75_)	51 (39–63)	44 (31–60)	0.003	54 (42–63)	48 (36–62)	0.017	52 (38–63)	48 (33–57)	0.037
Route of delivery, n (%)			0.355			0.609			0.629
Vaginal	151 (35.5)	28 (28.0)		80 (36.0)	99 (32.7)		147 (33.6)	32 (36.8)	
Elective cesarean section	157 (36.9)	42 (42.0)		79 (35.6)	120 (39.6)		170 (38.8)	29 (33.3)	
Nonelective cesarean section	117 (27.5)	30 (30.0)		63 (28.4)	84 (27.7)		121 (27.6)	26 (29.9)	
Prematurity, n (%)	26 (6.1)	6 (6.0)	0.965	12 (5.4)	20 (6.6)	0.572	23 (5.3)	9 (10.3)	0.070
Birth weight, kg, median (P_25_-P_75_)	3.2 (2.9–3.5)	3.2 (2.9–3.5)	0.515	3.2 (3.0–3.6)	3.2 (2.9–3.5)	0.052	3.2 (2.9–3.5)	3.2 (3.0–3.6)	0.193
Breastfeeding, mo, median (P_25_-P_75_)	6 (3–12)	6 (3–18)	0.168	6 (3–12)	6 (3–15)	0.631	6 (3–12)	6 (3–12)	0.235
Complementary feeding, mo, median (P_25_-P_75_)	6 (5–6)	6 (6–6)	0.707	6 (5–6)	6 (5–6)	0.878	66 (5–6)	6 (6–6)	0.887
Current smoking, n (%)									
Maternal	47 (11.1%)	19 (19.0)	0.031	26 (11.7)	40 (13.2)	0.611	54 (12.3)	12 (13.8)	0.707
Paternal	97 (22.8)	23 (23.0)	0.970	48 (21.6)	72 (23.8)	0.564	100 (22.8)	20 (23.0)	0.975
Familial atopy, n (%)									
Maternal	61 (14.4)	36 (36.0)	<0.0001	22 (9.9)	75 (24.8)	<0.0001	74 (16.9)	23 (26.4)	0.036
Paternal	38 (8.9)	17 (17.0)	0.018	15 (6.8)	40 (13.2)	0.017	40 (9.1)	15 (17.2)	0.024
Comorbidity, n (%)									
Asthma	NA	NA	NA	3 (1.4)	22 (7.3)	0.001	19 (4.3)	6 (6.9)	0.306
Allergic rhinitis	65 (15.3)	39 (39.0)	<0.0001	NA	NA	NA	76 (17.4)	28 (32.2)	0.002
Atopic dermatitis	43 (10.1)	22 (22.0)	0.001	17 (7.7)	48 (15.8)	0.005	NA	NA	NA

NA, not applicable; P_25_-P_75_, 25th percentile–75th percentile.

### 3.3. CS and allergic diseases

Through multivariate analysis, nonelective CS was identified as an independent factor associated with BA (odds ratio [OR]: 3.51, *P* = 0.026); so were maternal smoking (OR: 4.17, *P* = 0.004) and AR (OR: 5.93, *P* < 0.0001).

Table 3 shows that the associated factors of AR were the following: male (OR: 1.61, *P* = 0.049), maternal (OR: 2.71, *P* < 0.0001) or paternal (OR: 3.59, *P* < 0.0001) history of atopy, BA (OR: 3.86, *P* < 0.0001), and AD (OR: 4.84, *P* < 0.0001); the delivery route was not a significant associated factor (*P* > 0.05). Finally, although the elective CS showed a significant association with AD (*P* = 0.048), the route of delivery as a whole did not show an association (*P* = 0.082, data not shown in the table) and was therefore excluded from this model. The associated factor of AD was the past personal history of AR (OR: 4.13, *P* < 0.0001).

**Table 3.** Multivariate analysis of factors associated with the allergic diseases

	Bronchial asthma			Allergic rhinitis			Atopic dermatitis		
	aOR	95% CI	*P*	aOR	95% CI	*P*	aOR	95% CI	*P*
Elective cesarean section*	1.40	0.44–4.49	0.570	—	—	0.917	—	—	0.048
Nonelective cesarean section*	3.51	1.16–10.61	0.026	—	—	0.963	—	—	0.061
Sex, male	—	—	0.810	1.61	1.00–2.60	0.049	—	—	0.700
Age ≥60 mo	—	—	0.055	—	—	0.878	—	—	0.871
Birth weight <2.5 kg	—	—	0.814	—	—	0.616	—	—	0.496
Maternal smoking	4.17	1.56–11.13	0.004	—	—	0.157	—	—	0.793
Maternal atopy	—	—	0.195	2.71	1.60–4.61	<0.0001	—	—	0.390
Paternal atopy	—	—	0.515	3.59	1.91–6.75	<0.0001	—	—	0.355
Asthma	NA	NA	NA	3.86	2.14–6.95	<0.0001	—	—	0.716
Allergic rhinitis	5.93	2.51–13.99	<0.0001	NA	NA	NA	4.13	2.39–**7**.15	<0.0001
Atopic dermatitis	—	—	0.966	4.84	1.99–11.76	<0.0001	NA	NA	NA

Odds ratio (OR) obtained by binary logistic regression.

95% CI, 95% confidence interval; aOR, adjusted odds ratio; NA, not applicable.

* vs vaginally.

Table 4 shows that the CS route of delivery was not associated with the presence of asthma symptoms, rhinitis, or dermatitis during the previous year (*P* > 0.05).

**Table 4.** Multivariate analysis of factors associated with the allergic disease symptoms in the previous year

	Wheezing the previous year			Rhinitis the previous year			Dermatitis the previous year		
	aOR	95% CI	*P*	aOR	95% CI	*P*	aOR	95% CI	*P*
Elective cesarean section*	—	—	0.497	—	—	0.432	—	—	0.265
Nonelective cesarean section*	—	—	0.430	—	—	0.786	—	—	0.616
Sex, male	—	—	0.283	1.81	1.25–2.60	0.002	—	—	0.534
Age ≥60 mo	—	—	0.131	0.65	0.44–0.96	0.032	0.51	0.29–0.88	0.015
Birth weight <2.5 kg	—	—	0.820	—	—	0.392	—	—	0.093
Maternal smoking	2.23	1.20–4.13	0.011	—	—	0.743	—	—	0.450
Maternal atopy	2.72	1.62–4.56	<0.0001	2.96	1.75–5.02	<0.0001	—	—	0.148
Paternal atopy	—	—	0.379	—	—	0.196	—	—	0.171
Asthma	NA	NA	NA	5.63	1.61–19.69	0.007	—	—	0.476
Allergic rhinitis	2.70	1.56–4.56	<0.0001	NA	NA	NA	2.30	1.37–3.86	0.002
Atopic dermatitis	1.84	0.99–3.41	0.052	2.12	1.16–3.89	0.014	NA	NA	NA

Odds ratio (OR) obtained by binary logistic regression.

95% CI, 95% confidence interval; aOR, adjusted odds ratio; NA, not applicable.

* vs vaginally.

## 4. Discussion

Our study shows that nonelective CS is strongly associated with the prevalence of BA, but not with AR or AD. Regarding allergic disease symptoms, there were no associated factors found with the route of delivery. Among other important associations, we observed that maternal smoking was related to BA. Male sex and parental history of allergic disease were found among patients with AR. A child´s personal history of atopic disease was associated with each of the allergic diseases.

International recommendations indicate that the maximum number of CS births should not exceed 19%. Although this cutoff point has been associated with lower maternal and infant mortality [14], its role in atopic morbidity is still a matter of debate. Mexico is one of the leading countries where CS is performed (58.6%) [4], this led us to hypothesize that CS would be related to allergic diseases; however, we can only report an association between BA and CS, but not with AR, AD, or their symptoms. Recently, 4 independent meta-analyses showed that children born through CS have a higher risk of developing asthma than children born vaginally [15-18]. However, only one meta-analysis differentiated children delivered through elective CS vs urgent CS. In either case, an increased risk of asthma was observed [15]. On the other hand, several previous studies have not shown a relationship between CS and asthma or allergic diseases. In Switzerland, children born through either elective or urgent CS showed no greater risk of respiratory or allergic diseases than children born vaginally [11]. In Korea, the Cohort for Childhood Origin of Asthma and Allergic Diseases (COCOA) showed similar findings [9]. In Mexico, no association was observed between the route of delivery and allergic diseases and their symptoms [10]. Perhaps, no association has been correlated between CS and allergic diseases because prolonged and exclusive breastfeeding is capable of attenuating the risk [5, 7]. Various hypotheses have been put forward to explain the reasons why children born through CS seem to have a higher risk of developing allergic diseases; the most widely presumption has to do with changes in the neonatal microbiota. Children born through CS have fewer bacteria of the *Bifidobacterium* and *Bacteroides* genera, but an increased amount of bacteria of the *Clostridium*, *Lactobacillus*, *Enterobacter*, *Enterococcus*, and *Staphylococcus* genera [19]. Another theory has to do with the lower concentration of vitamin D observed in children born through CS [20]. Another key point relays the differences observed in the metabolome of children born through CS in whom fewer tryptophan, bile acid, and phenylalanine metabolites were observed compared with children born vaginally, which is reflected in an altered intestinal microbiota [21].

In our study, it was necessary to elucidate why only children born by nonelective CS had a higher risk of developing asthma than children born through elective CS and vaginally; perinatal medical care conditions and last-minute maternal functional changes are probably involved in its origin.

We report a maternal history of smoking was associated with BA and wheezing in the previous year. This finding has recently been documented in other parts of the world [22, 23]. Similarly, we demonstrate that children with a family history of atopy have a higher risk of developing allergic diseases; this event seems to be more relevant with a mother’s past medical history of atopy [24, 25].

Several limitations must be taken into consideration when interpreting our results. First, the cross-sectional nature of the study design did not allow for establishing causality; however, when investigating variables that change little or nothing over time such as the paternal history of allergic diseases, sex of the child, and delivery route, we can envision a certain degree of temporality. Second, the lack of verification of the diagnosis of allergic diseases in children, which is why questionnaires were included in the study. These questionnaires have been widely used in large epidemiological studies around the world. We must take into account that children who attend daycare centers are receiving continuous medical attention that allows for better timely detection of allergic diseases, as well as other diseases by medical personnel. Third, since the age of the study group was not uniform, there might be a possibility that there may have been children who, due to their young age, have not yet developed any of the allergic diseases. Fourth, children analyzed in this study represent a sample of children who attended public daycare centers in a single city, and results may vary by analyzing children who attended private daycare facilities and in other cities. Fifth, the survey did not delve into the reasons why CS was performed nonelectively. Finally, sixth, the characteristics of breastfeeding and the complementary introduction to the diet were not extensively analyzed either.

Children born through nonelective CS were most likely to develop BA, but not AR, or AD, when compared to children born vaginally. In the case of the symptoms of allergic diseases and the delivery route, we did not observe an association.

Other factors associated with the prevalence of BA were the current smoking status of the mother and a personal history of AR. Male sex, maternal or paternal history of atopy, and the personal history of BA and AD were associated with AR. As to AD, the only identified factor was a child´s personal history of AR.

## Acknowledgements

The authors sincerely thank the medical and administrative staff at DIF, as well as the parents of the children who participated in this study for their assistance. Their invaluable support and collaboration were essential for the successful completion of this research. We genuinely appreciate the facilities provided, their willingness to answer our questions, facilitate data access, and their commitment and patience throughout the study.

## Conflicts of interest

The authors have no financial conflicts of interest.

## Author contributions

Conception and design: Bedolla-Barajas and Gaxiola-de Alba. Provision of study materials or patients: Contreras-Aceves, Gaxiola-de Alba, and del Rocio Estrada-Bedolla. Analysis and interpretation of the data: Bedolla-Barajas, Morales-Romero; and Bedolla-Pulido. Statistical expertise: Morales-Romero. Critical revision of the article for important intellectual content and final approval of the article: Bedolla-Barajas, Morales-Romero; Contreras-Aceves, Gaxiola-de Alba, del Rocio Estrada-Bedolla, and Bedolla-Pulido.
